# Evolution of Liver Fibrosis in Romanian HCV Patients Following Treatment with Direct-Acting Antivirals

**DOI:** 10.3390/jcm14228112

**Published:** 2025-11-16

**Authors:** Alexia Anastasia Stefania Balta, Raisa Eloise Uibariu Barbu, Liliana Baroiu, Valentin Bulza, Florin Bujoreanu, Marius Moroianu, Mariana Daniela Ignat, Simona Claudia Cambrea, Elena Dumea, Valerian Stoian

**Affiliations:** 1 Faculty of Medicine & Pharmacy, ‘Dunărea de Jos’ University of Galați, 800008 Galați, Romaniamoroianu.g.marius@gmail.com (M.M.);; 2 Clinical Emergency County Hospital ‘Sf. Ap. Andrei’, 800578 Galati, Romania; 3 Clinical Children Emergency Hospital ‘Sf. Ioan’, 800487 Galati, Romania; 4 Clinical Hospital of Infectious Diseases ‘Sf. Cuv. Parascheva’, 800179 Galati, Romania; 5 General Hospital ‘CF’, 800223 Galati, Romania; 6 Faculty of Medicine & Pharmacy, Ovidius’ University of Constanta, 900527 Constanta, Romania; 7 Clinical Hospital of Infectious Diseases, 900178 Constanta, Romania; 8 National Institute of Public Health, 050463 Bucharest, Romania

**Keywords:** HCV, liver fibrosis, direct-acting antivirals

## Abstract

**Background/Objectives**: This study was conducted in an infectious disease clinic in southeastern Romania on a cohort of patients with chronic hepatitis C virus (HCV) infection. It aims to longitudinally evaluate biochemical parameters and the progression of liver fibrosis following treatment with direct-acting antivirals. **Methods**: One hundred eighty-one patients who experienced improvement in hepatic fibrosis 1–8 years after treatment (group A) were compared with eighty-six patients (except F0–F1) who had stable fibrosis or experienced worsening of hepatic fibrosis after antiviral treatment (group B). **Results**: The study demonstrated improvement and normalization of mean biochemical parameters in both groups, starting 4 weeks after initiation of therapy and remaining stable up to 5 years post-treatment. The only biochemical parameter that did not return to normal values was serum glucose, which showed elevated mean levels in both groups, with the highest values observed at 5 years post-treatment. Among all 267 patients, 181 (67.79%) showed improvement in hepatic fibrosis after DAA (direct-acting antivirals) therapy, 62 (23.22%) had stable fibrosis, and 24 (8.98%) experienced fibrosis worsening. Of those with improvement, 115 (63.53%) improved by one fibrosis stage, 49 (27.07%) by two stages, and 17 (9.39%) by three stages. **Conclusions**: This study highlights that more than half of Romanian HCV patients experienced regression of hepatic fibrosis and sustained normalization of most biochemical parameters up to 5 years after DAA treatment, confirming the long-term hepatic benefits of antiviral therapy.

## 1. Introduction

In 2022, the World Health Organization reported that following COVID-19, viral hepatitis and tuberculosis were the second leading causes of death from communicable diseases, accounting for approximately 1.3 million deaths [1].

Chronic hepatitis C remains a major public health concern, requiring ongoing and concerted efforts to achieve its elimination by 2030, as targeted by the World Health Organization.

Romania reported in 2023 a prevalence of 0.9% for active chronic hepatitis C virus infection, corresponding to an estimated number of 136,999 adults still presenting active viral replication—a significantly decreasing trend compared to data from 2006–2008 [2,3].

Liver fibrosis is a dynamic process in which chronic inflammation induces the synthesis of collagen and other extracellular matrix components, accompanied by hepatocellular necrosis [4,5,6,7]. Hepatic stellate cells are primarily responsible for producing these matrix elements [8,9]. Fibrosis progression involves excessive extracellular matrix deposition, leading to structural remodeling of glycoproteins, collagen, hyaluronic acid, and proteoglycans. The formation of incomplete subendothelial basement membranes and matrix accumulation in the space of Disse (perisinusoidal fibrosis) ultimately disrupt hepatic microcirculation and impair hepatocyte function and clearance capacity [10].

In chronic HCV infection, persistent inflammation and injury at the hepatocyte level activate hepatic stellate cells and their transformation into myofibroblasts. Myofibroblasts also produce cytokines and enzymes with a role in fibrogenesis in the regulation of collagen and matrix catabolism (matrix metalloproteinases and tissue inhibitors of matrix metalloproteinases). Therefore, myofibroblasts play an essential role in the degradation of the extracellular matrix, a process known as fibrolysis.

Fibrogenesis develops when the activity of cytokines that stimulate cell regeneration exceeds that of inhibitory cytokines, leading to an imbalance between matrix metalloproteinases and their tissue inhibitors. Regenerative cytokines include hepatocyte growth factor, transforming growth factor-α, and epidermal growth factor. Citokines with pro-fibrogenic effects include platelet-derived growth factor, transforming growth factor-β1 (TGF-β1), and heparin-binding growth factor [11].

TGF-β1 is a key mediator of hepatic fibrogenesis, promoting stellate cell activation in myofifroblasts, extracellular matrix deposition, and hepatocyte apoptosis [12]. The resulting perisinusoidal fibrosis increases vascular resistance and contributes to portal hypertension and hepatocyte hypoxia [13]. However, fibrosis may partially regress through matrix remodeling mediated by matrix metalloproteinases [5,7,14,15].

Regression of hepatic fibrosis has been observed both in experimental studies and in animal models, with remodeling of the vascular architecture as well as improvement in hepatic lobular architecture [16].

At the cellular level, fibrosis regression primarily involves the inactivation of hepatic stellate cells once the injurious stimulus ceases, leading these cells to revert to a quiescent state or undergo senescence or apoptosis [17]. This process results in the inactivation of myofibroblasts, the activation of collagenases, and the subsequent degradation of the fibrillar extracellular matrix, accompanied by the resorption of fibrous septa within the liver [18].

The intrinsic capacity of the liver to regenerate may explain the lengthy period in which hepatic fibrosis progresses to the final stages of disease. The cirrhotic liver loses its regenerative ability after removal of the injurious factor. The more advanced the degree of fibrosis—especially in hepatic cirrhosis—the lower the chance of fibrosis regression. Thus, once important architectural changes, vascular collapse, and portal hypertension occur, it is considered that the collagen bands formed cannot be destroyed by normal collagenases, and the progression of liver disease cannot be stopped [19].

In addition to achieving SVR (Sustained Viral Response), genetic predisposition, alcohol consumption, inflammation linked to hepatic steatosis, or the presence of diabetes mellitus (DM) are also implicated in the progression or maintenance of fibrosis [20].

Liver fibrosis is an intermediate and essential stage in the progression to cirrhosis, its stage impacting the patient’s prognosis and establishing the time interval for monitoring patients post-therapy [2,21,22,23,24,25].

Clinical studies have observed, in patients with SVR after DAA, the regression of fibrosis, the reduction of overall and hepatic-related mortality, a lower rate of hepatocellular carcinoma (HCC), and the improvement and reduction of the incidence of extrahepatic manifestations associated with HCV infection [26,27].

Our study aims to evaluate fibrosis regression after SVR post-DAA, assessed by Fibroscan, an important factor impacting the prognosis of patients with chronic HCV infection.

## 2. Materials and Methods

We present a prospective observational study of adult patients with chronic HCV infection, treated with DAA therapies according to the national protocols applicable in Romania during the period of 2017–2024 and monitored at the 2nd Clinic of the “Sf. Cuv. Parascheva” Clinical Infectious Diseases Hospital, Galați. The study was approved by the Ethics Committee of the “Dunărea de Jos” University of Galați, no. 42426 from 4 December 2024, and all patients signed informed consent before inclusion in the analysis.

Patients underwent initial biochemical evaluation, abdominal ultrasound, and comorbidity screening, and if necessary, they underwent assessment of drug interactions; hepatic fibrosis evaluation by Fibroscan or Fibromax; biochemical assessments at 1 month, 2 months, 3 months, 6 months, 12 months, 36 months, and 60 months after starting antiviral treatment; HCV RNA (hepatitis C virus ribonucleic acid) at the beginning and 12 weeks after the end of antiviral therapy; and an evaluation of hepatic fibrosis by Fibroscan between 1 January 2025 and 31 July 2025 (performed at least 12 months after starting antiviral treatment).

Information was collected from the patients’ observation charts regarding demographic parameters (age, sex, area of origin) and a complete medical history for all patients and biochemical parameters: complete blood count (CBC), Aspartate aminotransferase (AST), Alanine aminotransferase (ALT), direct and total bilirubin, blood glucose, albumin, amylase, urea, creatinine, international normalized ratio (INR), prothrombin concentration, alpha-fetoprotein (AFP), HCV RNA (only at the initial visit and at the end of antiviral therapy), abdominopelvic ultrasound and liver fibrosis (at the initial visit), and a Fibroscan evaluation in 2025 [28].

Patients predominantly had HCV genotype 1 infection, accounting for 99% of circulating strains in Romania [29], were of white race, had Romanian ethnicity, and received antiviral therapies with various regimens according to the indications of the national program at the time of therapy initiation, namely ombitasvir/paritaprevir/ritonavir and dasabuvir, ledipasvir/sofosbuvir, elbasvir/grazoprevir, sofosbuvir/velpatasvir, or glecaprevir/pibrentasvir [30].

Inclusion criteria were as follows:
   i. Adult patients with viremic HCV infection, regardless of the degree of fibrosis, who underwent DAA therapy in our clinic and achieved SVR.
  ii. Patients who signed informed consent.
Exclusion criteria were as follows:
    i. Patients under the age of 18.
   ii. Patients who refused to sign or were unable to sign the informed consent for participation in the study.
  iii. Patients meeting exclusion criteria for starting antiviral therapy (active neoplasms or decompensated cirrhosis Child B or C who received antiviral therapy in a gastroenterology clinic).
  iv. Patients who did not achieve SVR after the first course of DAA and were subsequently treated with Sofosbuvir/Velpatasvir/Voxilaprevir as well as patients who obtained SVR and were reinfected with HCV and underwent a second course of DAA.
    v. Patients who interrupted the DAA course due to adverse effects.
   vi. Patients who did not present in 2025 for the hepatic fibrosis evaluation visit.
  vii. Patients with initial fibrosis F0–F1 (73 patients), 64 of whom maintained their fibrosis after antiviral treatment and 9 patients who worsened it possibly due to factors independent of HCV.

Regarding patient groups, of the total of 714 patients treated in our clinic, 340 came to the fibrosis assessment visit in 2025, and 267 met all the inclusion criteria in the study and were divided into the study groups as follows:

(1) Group A—181 patients who experienced improvement in hepatic fibrosis in 2025 compared to initial fibrosis;
(2) Group B—86 patients who had stable fibrosis or experienced worsening of hepatic fibrosis (except patients with initial fibrosis F0–F1).

The comparative analysis was conducted in groups A and B.

Statistical analyses were performed using IBM SPSS^®^ Statistics version 22 (IBM Corp New York, NY, USA). For each continuous variable, descriptive analysis was performed, with mean and standard deviation (SD), and for categorical variables, absolute frequency was used. For comparisons, Student’s *t*-test and Chi-Square (χ^2^) test were used. The difference between means and percentages with a *p*-value under 0.05 was considered statistically significant.

The interpretation values of liver fibrosis were as follows: 2–7 kPa for F0–F1, 7.1–9.4 kPa for F2, 9.5–12.4 kPa for F3, and ≥ 12.5 kPa for F4 [31]. The normal values for biochemical analyses were as follows: ALT-0–45 U/L, AST-0/35 U/L, total bilirubin (TBR)-0.1–1.2 mg/dL, direct bilirubin (DBR)-0–0.2 mg/dL, total leukocyte count 4000–10,000/ μL, hemoglobin 13–17 g/dL, platelets-150–450 *×* 10^3^/μL, INR-0.80–1.20, serum albumin (ALB)-3.5–5.5 g/dL, AFP-1–10 UI/mL, urea 21–43 mg/dL, creatine 0.8–1.3 mg/dL, serum amylase 0–100 U/L, serum glucose 74–106 mg/dL, prothrombin serum concentration 70–130%, and gamma glutamate transpeptidase (GGT) 0–55 U/L.

## 3. Results

A comparative analysis of group A, comprising 181 patients who experienced improvement in hepatic fibrosis following antiviral DAA treatment, with group B, comprising 86 patients who, after this therapy, maintained their level of fibrosis or experienced its worsening, revealed the following:

- Females predominated in both groups (*p* = 0.1705, without relevant statistical significance) (Table 1).
- The average age in group A was 60.2 years, while in group B, it was 59.96 years, without relevant statistical significance (*t*-test, *p* = 0.8645) (Table 1).
- At the initial assessment, the two groups did not present statistically significant differences in the means of the biochemical parameters ALT, AST, total and direct bilirubin, serum creatinine, serum glucose, serum albumin, the prothrombin concentration, and the INR. The average platelet count, total leucocyte count, and hemoglobin are lower in group B, statistically significant, compared to group A (Table 2). At the initiation of therapy, hepatic fibrosis predominantly in both groups was F2 (Table 3).

Homogenization of groups by propensity score was as follows.

It is observed that there were inhomogeneous variables between the two groups (there were statistically significant differences between the two groups). In order to analyze the end point (the evolution of liver fibrosis post-DAA), it was decided to “attenuate” these differences using a propensity score. The propensity score was calculated using the R twang package version 1.5.: Toolkit for Weighting and Analysis of Nonequivalent Groups [32], the propensity score being calculated using an ATE (“average treatment effect”)-type algorithm. To calculate the score, the variables that revealed statistically significant differences in the tests were taken into account. The propensity score calculation algorithm was a “general boosting” type based on regression trees and included 10,000 iterations to ensure that the attenuation by the propensity score was optimal. After attenuation with propensity scores, a significant overlap of the two groups was obtained, the statistically significant differences existing before attenuation being “transformed” in their vast majority into differences without statistical significance.

At 3 months after antiviral therapy, the two groups did not present statistically significant differences in the means of the biochemical parameters: total bilirubin, total leucocyte count, platelet count, prothrombin concentration, serum hemoglobin, and serum creatinine. The average of ALT, AST, the INR, and serum glucose was statistically significantly higher in group B (Table 4).

The 12-month evaluation following the initiation of antiviral therapy does not reveal any statistically significant differences between the groups in terms of the averages of total bilirubin, direct bilirubin, hemoglobin, serum creatinine, amylase, and albumin. The average of the serum amylase, prothrombin concentration, total leukocyte count, and platelet count ware statistically significantly higher in group A, but the average values in both groups were within the normal limits of the test. The average of the serum glucose was statistically significantly higher in group B, and the average values were higher than the normal limits of the test. The average of the ALT, AST, the INR, and AFP were statistically significantly higher in group B, but the average values in both groups were within the normal limits of the test (Table 5).

At the 60-month evaluation after the initiation of antiviral therapy, the average serum creatinine is statistically significantly higher in group A than in group B, but both are within the normal range for the test. The averages of AST and total leukocyte count were statistically significantly higher in group B, but the average values in both groups were within the normal limits of the test. The average of the serum glucose was highter than the normal limits of the test in the both groups, without statistically significant differences (Table 6).

A decrease in the average values of ALT and AST is observed in both groups after one month of treatment, with their levels remaining within normal limits up to five years. The mean levels of ALT and AST, although lower than the maximum normal value of the test, remain higher, statistically significantly, in group B at 3 and 12 months and that of AST at 5 years.

Regarding cholestasis, the average values of both direct and total bilirubin in both groups showed a decrease two months after starting treatment and maintained low values, within the normal laboratory range, up to five years after the initiation of antiviral therapy. At 3 months, a statistically significant higher average level of direct bilirubin was noted in group B, which indicates a more delayed normalization of direct bilirubin in the group with maintenance or worsening of fibrosis.

Regarding the average serum levels of leukocytes, platelets, and hemoglobin, they remain within normal values during the 5 years of monitoring, and we note higher average values of these three parameters at the initial evaluation, statistically significant, in group A, which allows for a prediction of the favorable evolution of fibrosis in patients with these higher parameters. We also note statistically significantly higher mean values of leukocytes and platelets in group A at 12 months, with a reversal at 5 years, with higher mean leukocytes in group B.

Regarding hepatic support of coagulation function, quantified by mean INR values, we note statistically significant higher values at 3 and 12 months in group B, which can be considered a predictive factor of the unfavorable evolution of liver fibrosis in these patients, after antiviral treatment.

Monitoring the protein secretion function by the mean values of serum albumin in the two groups reveals slight increases at 12 and 60 months, which may support the idea of slow, progressive functional hepatic recovery up to 5 years after the start of antiviral therapy.

The mean glycemic values in both groups were above the normal range for this test, indicating the presence of diabetic patients in both groups. The variations in mean glucose values during the follow-up period were slight, but at five years, there was a tendency for these values to increase, which highlights the need for closer monitoring of glycemic control in patients with a history of chronic HCV hepatitis over the long term.

However, we note statistically significantly higher mean blood glucose values in group B at 3 and 12 months after antiviral treatment, which highlights the negative impact of hyperglycemia on the progression of liver fibrosis.

Fibrosis evaluation conducted between 1 and 8 years after the start of DAA antiviral therapy showed statistically significant differences in the distribution of all fibrosis stages between the two groups, indicating that the majority of patients who improve their fibrosis after DAA remain in F0–F1, and patients who maintain or worsen their fibrosis post-DAA are approximately equally divided between the F2, F3, and F4 fibrosis groups (Table 7).

**Figure 1:**
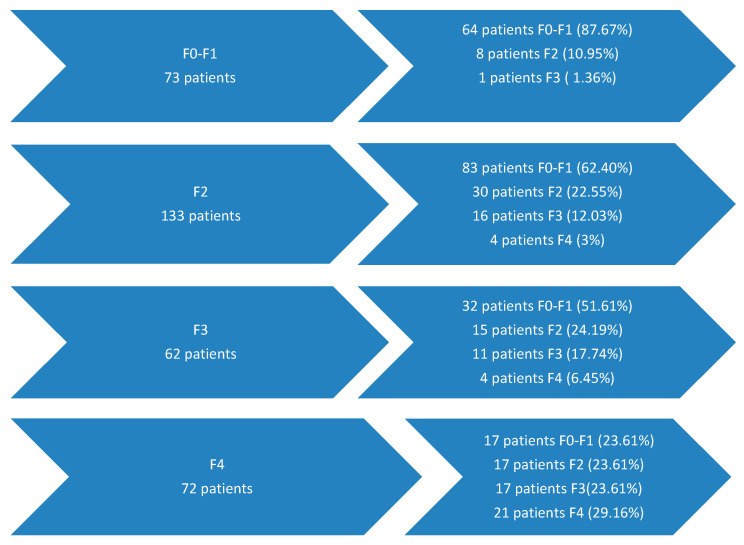
Liver fibrosis evolution 1–8 years after antiviral treatment with DAAs.

The evaluation of fibrosis in the total group at an interval ranging from 1 to 8 years after the start of antiviral treatment with DAA revealed that the majority of patients who begin this treatment at the early stage of the disease (F0–F1) maintain this initial stage of fibrosis in 87.67% of cases. Most patients with moderate fibrosis (F2) (62.4%) improve their fibrosis to F0–F1, and in 22.55% of cases, they maintain the same degree of fibrosis. Among patients with advanced fibrosis (F3), about half manage to progress to minimal fibrosis (F0–F1) after treatment (51.61%), and about a quarter progress to moderate fibrosis (F2)—24.19%. Patients with hepatic cirrhosis manage to improve their fibrosis in 70.83% of cases (Figure 1).

From another perspective, out of the total group, 181 patients (53.23%) improved their degree of hepatic fibrosis after DAA treatment, 126 patients (37.05%) maintained constant fibrosis, and 33 patients (9.70%) experienced worsening of fibrosis after treatment.

Of the 181 patients who experienced improvement in fibrosis, 115 patients (63.53%) improved hepatic fibrosis by one grade, 49 patients (27.07%) improved by two grades, and 17 patients (9.39%) improved by three grades.

## 4. Discussion

Today, we have at our disposal many studies that prove the importance of treating HCV infection as early as possible. The impact on liver fibrosis is extremely significant in the cascade toward liver cirrhosis, portal hypertension, liver carcinoma, quality of life, and death. Demonstrating the impact of DAA therapies on liver fibrosis is very important in overcoming logistical and financial barriers for implementing screening in the general population for HCV and other measures suggested by the WHO to meet the eradication targets for 2030.

Our study notes the improvement and normalization of the average biochemical parameters both in the group of patients who experienced improvement in liver fibrosis post-treatment and in those with maintenance or worsening of liver fibrosis post-antiviral treatment starting with the assessment 4 weeks after the start of treatment and maintained constant until the assessment 5 years post-treatment. The only biochemical parameter whose average did not fall within normal values was serum glycemia, which showed increases in both groups, with the highest average values at the assessment 5 years post-treatment. This increase may be correlated with the increase in the incidence of diabetes mellitus in the general population in elderly patients (the average age in both groups being over 60 years at 5 years after antiviral treatment) and with the fact that the hepatoprotective diet does not impose restrictions on the daily amount of carbohydrates. Also, significantly higher mean blood glucose values at 3 and 12 months after DAA treatment in patients who experienced maintenance or worsening of liver fibrosis underline the negative impact of metabolic factors, namely hyperglycemia, on the evolution of liver fibrosis. This observation requires larger studies related to the prevalence of hyperglycemia in patients with a history of chronic liver pathology, multidisciplinary monitoring of these patients, and possibly personalized diets and early therapy of metabolic pathology.

Regarding the average serum levels of leukocytes, platelets, and hemoglobin, they remain within normal values during the 5 years of monitoring, and we note statistically significant higher average values of these three parameters at the baseline in group A, which allows for a prediction of the favorable evolution of fibrosis in patients with these higher parameters, after DAA.

Regarding coagulation function, quantified by mean INR values, we note statistically significant higher values at 3 and 12 months in group B, which can be considered a predictive factor of the unfavorable evolution of liver fibrosis in these patients, after DAA.

In the era of HCV therapy with interferon, the SVR rate was much lower and the rate of adverse reactions much higher than during DAA therapies. Most evidence for monitoring the regression of liver fibrosis after SVR through liver biopsy comes from the era of interferon therapies.

One example is the study by Poynard et al., 2002, which evaluated the impact of Peg-interferon and ribavirin therapy on liver fibrosis through serial liver biopsies and observed, in patients with SVR, histological improvement of fibrosis in 55% of patients [33].

George et al. (2009) reported fibrosis regression in 81.5% of patients five years after achieving SVR with pegylated interferon and ribavirin [34]. Although liver biopsy remains the diagnostic standard, non-invasive methods such as FibroScan and transient elastography are now preferred for their safety and simplicity. However, transient elastography may overestimate fibrosis regression after HCV eradication, as liver stiffness can decrease rapidly with the resolution of inflammation following DAA therapy.

A study involving 209 patients who achieved SVR following direct-acting antiviral therapy assessed liver fibrosis using FibroScan three years after SVR. The results showed fibrosis improvement in 57% of patients, fibrosis progression in 7%, and stable fibrosis in 36% of cases [35].

Another study evaluating liver stiffness after achieving SVR demonstrated a gradual but statistically significant decrease over time: from 19.4 ± 12.9 kPa at treatment initiation to 13.9 ± 9.1 kPa at 48 weeks, 11.7 ± 8.2 kPa at 96 weeks, and 10.09 ± 6.23 kPa at 144 weeks (*p* < 0.001) [36].

In our study, which assessed changes in liver fibrosis 1–8 years after the initiation of antiviral therapy using FibroScan, 181 patients (53.23%) showed improvement in fibrosis stage, 126 patients (37.05%) had stable fibrosis, and 33 patients (9.70%) exhibited fibrosis progression.

These findings are consistent with previous reports [35] and further emphasize the beneficial impact of DAA therapy on liver fibrosis. By slowing the progression to decompensated cirrhosis and hepatocellular carcinoma, DAA treatment contributes to improved long-term outcomes and enhanced quality of life in affected patients.

The worsening of liver fibrosis after successful antiviral treatment (completed with undetectable HCV RNA and improvement of biochemical parameters of liver function) may be attributed to an initial advanced degree of liver fibrosis; the presence of HBV (Hepatitis B Virus) and HDV (Hepatitis D Virus) coinfections; and comorbidities such as hepatic steatosis, obesity, dyslipidemia, atherosclerosis, diabetes mellitus, hypertension, heart failure, genetic predisposition, and the consumption of alcohol or potentially hepatotoxic medications [37,38,39,40].

Clinical trial observations note a positive impact of SVR post-DAA on long-term overall mortality [41], a reduction in portal hypertension and variceal bleeding post-DAA [42], and a reduction in the risk of liver carcinoma [43], but still with the need for annual monitoring, especially in patients with advanced residual liver fibrosis. Extrahepatic manifestations of chronic HCV infection improve and decrease in incidence in patients with post-DAA SVR [3].

The limitations of this study stem from the differing methods of liver fibrosis assessment at therapy initiation—either by Fibromax or Fibroscan; from the follow-up evaluation with Fibroscan performed in approximately half of the treated patients in the clinic, specifically those who presented for evaluation, likely representing the group with the highest compliance to post-treatment monitoring; and from the relatively small size of the patient cohort. Another limitation of this study is the high drop-out rate observed (374 patients), which may have impacted the accuracy of the reported rate of fibrosis progression. This factor should be considered when interpreting the findings.

The antiviral treatment was conducted in accordance with the national treatment protocol for DAA regimens. Although the study did not aim to compare specific therapeutic schemes, the observed trends indicate a satisfactory regression of fibrosis over time following antiviral therapy. The findings are consistent with previously reported outcomes in patients achieving sustained virological response, supporting the beneficial long-term impact of DAA treatment on liver fibrosis.

The strengths of the study lie in the fact that it provides data on liver fibrosis monitoring between 1 and 8 years after the initiation of DAA therapy—a relatively new treatment, introduced in 2017 in our clinic—with few studies in the literature assessing the long-term impact of these therapies, both in terms of liver fibrosis and in maintaining post-treatment hepatic functional balance.

## 5. Conclusions

Our study observed the improvement and normalization of all mean biochemical parameters of liver function 5 years after DAA treatment, the increase in mean serum glucose values exceeding normal values 5 years after DAA, the improvement of the degree of liver fibrosis (assessed by Fibroscan 1–8 years after antiviral therapy) in 53.23% of patients, and the maintenance of the degree of fibrosis in 37.05% of patients. Our study observed that higher leukocyte, platelet, and hemoglobin values at the start of antiviral treatment may be predictive of improvement in post-treatment fibrosis and that higher INR values at 3 and 12 months post-treatment may be predictive of maintenance or worsening of post-DAA fibrosis are in agreement with observations from other studies that described lower improvements in fibrosis in patients with long-standing infection and advanced stages of fibrosis.

Direct-acting antiviral therapy for hepatitis C virus infection demonstrates sustained long-term efficacy, leading to improvement and stabilization of hepatic biochemical function and reduction of liver fibrosis in more than half of treated patients. Over time, this therapy has a beneficial impact on both patients’ quality of life and overall prognosis.

## Figures and Tables

**Table 1:** The analysis of the age and sex of patients in the two groups.

		Group A		Group B	
		Mean (SD)	Count, %	Mean (SD)	Count, %
Patient age at the initiation of antiviral therapy (years)		60.2 (10.94)		59.96 (10.27)	
Patients’ sex	Masculine		63 (34.80%)		22 (25.58%)
	Feminine		118 (65.19%)		64 (74.41%)

**Table 2:** Baseline characteristics of patients in group A and group B.

	N = 181 (Group A)			N = 86 (Group B)			p (*t*-Test)
	Av.	SD	Median	Av.	SD	Median	
Leukocytes (N/µL)	6.95	1.85	6.60	6.23	1.84	6.03	**0.0032**
Hb (g/dL)	14.55	1.43	14.60	13.79	1.99	14	**0.0004**
Platelets (N/µL)	214.77	67.95	204	202.41	73.15	199	**0.0014**
ALT (U/L)	85.56	72.01	60.50	84.44	62.25	70.25	0.9015
AST (U/L)	68.47	57.57	49	74.42	53.59	63.95	0.4206
Total bilirubin (mg/dL)	0.78	0.32	0.70	0.83	0.37	0.75	0.2581
Direct bilirubin (mg/dL)	0.3	0.15	0.27	0.35	0.27	0.29	0.0533
Prothrombin concentration (%)	94.66	15.12	93.7	91.51	17.95	93.35	0.1360
Serum albumin (g/dL)	4.55	0.34	4.54	4.47	0.27	4.49	0.0568
Serum creatinine (mg/dL)	0.84	0.18	0.81	0.83	0.21	0.81	0.6883
Serum glucose (mg/dL)	113.52	19.09	104.1	116.31	42.12	105.2	0.4561
INR	1.02	0.92	1	1.02	0.14	1.02	1
RNAHCV (UI/mL)	1,742,977.95	2,499,433.34	22,200,000	1,892,322.73	2,534,584.15	926,270.5	0.6501

Abbreviations: Av., Average; SD, standard deviation; T-*t*, Student’s t-test (*t*) for differences between means.

**Table 3:** Liver fibrosis at the initiation of antiviral therapy.

		Group A (181 Patients)		Group B (86 Patients)		*p*-Value
		No	%	No	%	
**Initial stage of fibrosis in patients from the studied groups**	F0–F1	0	0.0%	0	0%	1
	F2	83	45.85%	50	58.13%	0.0811
	F3	47	25.96%	15	17.44%	0.1658
	F4	51	28.17%	21	24.41%	0.6175

**Table 4:** Clinical parameters at 3 months among patients in group A and group B.

	N = 181 (Group A)			N = 86 (Group B)			*p* (*t*-Test)
	Av.	SD	Median	Av.	SD	Median	
Leukocytes (N/µL)	6.93	2.22	6.5	6.66	1.57	6.65	0.3118
Platelets (N/µL)	220.64	60.85	219.5	204.85	62.26	194	0.0503
Hb (g/dL)	14.42	1.16	14.50	14.13	1.20	1396	0.0602
ALT(U/L)	22.44	9.82	19.75	27.43	12.55	26.7	**0.0005**
AST(U/L)	24.54	8.09	22.15	28.87	9.17	27.2	**0.0001**
Total bilirubin (mg/dL)	0.67	0.33	0.59	0.74	0.41	0.63	0.1362
Direct bilirubin (mg/dL)	0.20	0.09	0.19	0.26	0.19	0.21	**0.0005**
Prothrombin concentration (%)	97.77	17.24	98.2	94.34	20.9	94.6	0.1579
Serum albumin (g/dL)	4.53	0.34	4.54	4.46	0.27	4.49	0.0952
Serum creatinine (mg/dL)	0.87	0.19	0.85	0.84	0.23	0.80	0.2618
Serum glucose (mg/dL)	108.56	19.09	104.1	118.16	42.12	105.2	**0.0109**
INR	1.01	0.11	1	1.05	0.15	1.03	**0.0146**

**Table 5:** Clinical parameters at 12 months after initiation of antiviral therapy (group A vs. group B).

	N = 181 (Group A)			N = 86 (Group B)			*p* (*t*-Test)
	Av.	SD	Median	Av.	SD	Median	
Leukocytes (×10^6^/µL)	6.56	2.02	6.2	7.1	1.92	6.95	**0.0391**
Platelets (×10^3^/µL)	219.35	65.92	216	190.43	63.15	195.5	**0.0008**
Hb (g/dL)	14.11	1.19	14	14.04	1.66	13.9	0.6943
ALT (U/L)	21.26	8.85	19.3	26.77	9.62	26.2	**<0.0001**
AST (U/L)	24.12	6.66	22.8	26.88	7.67	27.2	**0.0029**
Total bilirubin (mg/dL)	0.71	0.39	0.6	0.72	0.44	0.6	0.8512
Direct bilirubin (mg/dL)	0.21	0.09	0.19	0.23	0.16	0.18	0.1933
Prothrombin concentration (%)	99.0	15.06	97.05	89.6	15	88.45	**<0.0001**
Serum albumin	4.58	0.29	4.62	4.56	0.33	4.58	0.6152
Serum creatinine (mg/dL)	0.85	0.16	0.83	0.85	0.24	0.79	1
Serum glucose	107.1	20.19	102.6	127.95	59.28	108.85	**<0.0001**
INR	1.01	0.08	1.01	1.06	0.1	1.05	**<0.0001**
AFP (ng/mL)	3.05	2.18	2.43	3.75	1.98	3.23	**0.0180**
Amylase (U/L)	91.38	39.54	84.1	88.23	44.18	84.6	**<0.0001**

**Table 6:** Comparison of clinical and laboratory parameters between group A and group B at 5 years following initiation of antiviral therapy.

	N = 181 (Group A)			N = 86 (Group B)			*p* (*t*-Test)
	Av.	SD	Median	Av.	SD	Median	
Leukocytes (×10^6^/µL)	6.52	1.93	5.75	6.85	1.78	6.75	**0.0156**
Platelets (×10^3^/µL)	210.28	67.9	216	214.21	70.68	207.5	0.6631
Hb (g/dL)	14.1	1.43	14.05	14.06	1.05	13.85	0.8172
ALT (U/L)	25.55	8.99	22.65	27.53	11.97	25.55	0.1334
AST (U/L)	26.35	7.86	26	29.02	11.01	28.35	**0.0242**
Total bilirubin (mg/dL)	0.78	0.39	0.7	0.6	0.16	0.54	**0.0001**
Direct bilirubin (mg/dL)	0.25	0.09	0.22	0.24	0.18	0.20	0.5733
Prothrombin concentration (%)	100.32	15.5	97	99.67	11.52	98.6	0.7296
Serum albumin (g/dL)	4.62	0.36	4.65	4.62	0.34	4.61	1
Serum creatinine (mg/dL)	0.89	0.19	0.87	0.77	0.14	0.78	**<0.0001**
Serum glucose (mg/dL)	113.77	37.1	110	118.67	25.51	116.3	0.2696
INR	1.01	0.12	1.02	1.01	0.01	1.01	1
AFP (ng/mL)	3.12	1.66	2.8	3.37	1.7	2.76	0.2549
Amylase (U/L)	90.17	34.24	79.1	93.46	38	85.15	0.4797

**Table 7:** Hepatic fibrosis 1–8 years after the initiation of antiviral therapy.

		Group A (181 Patients)		Group B (86 Patients)		*p*-Value
		No	%	No	%	
**Fibrosis stage-1–8 years after antiviral therapy in patients from the studied groups**	F0–F1	132	72.92%	0	0%	**<0.0001**
	F2	32	17.67%	30	34.88%	**0.0031**
	F3	17	9.39%	27	31.39%	**<0.0001**
	F4	0	0%	29	33.72%	**<0.0001**

## Data Availability

Data Availability Statements are available on request through the corresponding author.

## References

[1] World Health Organization Global Hepatitis Report 2024. https://www.who.int/publications/i/item/9789240091672.

[2] Centrul Național de Supraveghere și Control al Bolilor Transmisibile Hepatita Virală Tip B și C—Analiza Rezultatelor Studiului Sero-Epidemiologic de Prevalență, România, Anii 2022–2023. https://www.cnscbt.ro/index.php/analiza-date-supraveghere/hepatita-virala-tip-b-si-c.

[3] Balta A.A.S., Ignat M.D., Barbu R.E., Dumitru C., Radaschin D.S., Bulza V., Mateescu Costin S.A., Pleșea-Condratovici C., Baroiu L. (2025). Impact of Direct-Acting Antivirals on Extrahepatic Manifestations in Chronic Hepatitis C: A Narrative Review with a Hermeneutic Approach. Healthcare.

[4] World Health Organization (2023). Hepatitis C.

[5] World Health Organization Global Health Sector Strategy on Viral Hepatitis 2016–2021. https://www.bing.com/search?q=5.+World+Health+Organization.+Global+Health+Sector+Strategy+on+Viral+Hepatitis+2016-2021%3A+Towards+Ending+Viral+Hepatitis.+WHO%2C+June+2016%2C+pp.+1-56&cvid=41472bfe1fff44168cd6d7f0852a9897&gs_lcrp=EgRlZGdlKgYIABBFGDkyBggAEEUYOTIHCAEQ6wcYQNIBBzYzMWowajmoAgiwAgE&FORM=ANAB01&PC=NMTS.

[6] Friedman S.L. (2010). Evolving challenges in hepatic fibrosis. Nat. Rev. Gastroenterol. Hepatol..

[7] Hernandez-Gea V., Friedman S.L. (2011). Pathogenesis of liver fibrosis. Annu. Rev. Pathol..

[8] World Health Organization (2017). Global Hepatitis Report 2017.

[9] Pinzani M. (2015). Pathophysiology of liver fibrosis. Dig. Dis..

[10] Gressner O.A., Weiskirchen R., Gressner A.M. (2007). Biomarkers of liver fibrosis: Clinical translation of molecular pathogenesis or based on liver-dependent malfunction tests. Clin. Chim. Acta.

[11] Henderson N.C., Iredale J.P. (2007). Liver fibrosis: Cellular mechanisms of progression and resolution. Clin. Sci..

[12] Gressner A.M., Weiskirchen R., Breitkopf K., Dooley S. (2002). Roles of TGF-β in hepatic fibrosis. Front. Biosci..

[13] Gressner A.M., Weiskirchen R. (2003). The tightrope of therapeutic suppression of active transforming growth factor-beta: High enough to fall deeply?. J. Hepatol..

[14] Thomas D.L. (2013). Global control of hepatitis C: Where challenge meets opportunity. Nat. Med..

[15] Seki E., Brenner D.A. (2015). Recent advancement of molecular mechanisms of liver fibrosis. J. Hepatobiliary Pancreat. Sci..

[16] Zoubek M.E., Trautwein C., Strnad P. (2017). Reversal of liver fibrosis: From fiction to reality. Best Pract. Res. Clin. Gastroenterol..

[17] Troeger J.S., Mederacke I., Gwak G.Y., Dapito D.H., Mu X., Hsu C.C., Pradere J.P., Friedman R.A., Schwabe R.F. (2012). Deactivation of hepatic stellate cells during liver fibrosis resolution in mice. Gastroenterology.

[18] Povero D., Busletta C., Novo E., Di Bonzo L.V., Cannito S., Paternostro C., Parola M. (2010). Liver fibrosis: A dynamic and potentially reversible process. Histol. Histopathol..

[19] Khan S.M.D., Saxena R.M. (2021). Regression of hepatic fibrosis and evolution of cirrhosis: A concise review. Adv. Anat. Pathol..

[20] Hedenstierna M., Nangarhari A., El-Sabini A., Weiland O., Aleman S. (2018). Cirrhosis, high age and high body mass index are risk factors for persisting advanced fibrosis after sustained virological response in chronic hepatitis C. J. Viral Hepat..

[21] Manns M.P., McHutchison J.G., Gordon S.C., Rustgi V.K., Shiffman M., Reindollar R., Goodman Z.D., Koury K., Ling M., Albrecht J.K. (2001). Peginterferon alfa-2b plus ribavirin compared with interferon alfa-2b plus ribavirin for initial treatment of chronic hepatitis C: A randomised trial. Lancet.

[22] McHutchison J.G., Gordon S.C., Schiff E.R., Shiffman M.L., Lee W.M., Rustgi V.K., Goodman Z.D., Ling M.H., Cort S., Albrecht J.K. (1998). Interferon alfa-2b alone or in combination with ribavirin as initial treatment for chronic hepatitis C. N. Engl. J. Med..

[23] Sharma S., Khalili K., Nguyen G.C. (2014). Non-invasive diagnosis of advanced fibrosis and cirrhosis. World J. Gastroenterol..

[24] AASLD-IDSA HCV Guidance: Recommendations for Testing, Management, and Treating Hepatitis C. When and in Whom to Initiate HCV Therapy. https://www.hcvguidelines.org/.

[25] Chou R., Wasson N. (2013). Blood tests to diagnose fibrosis or cirrhosis in patients with chronic hepatitis C virus infection: A systematic review. Ann. Intern. Med..

[26] Rockey D.C., Friedman S.L. (2021). Fibrosis regression after eradication of hepatitis C virus: From bench to bedside. Gastroenterology.

[27] Elsharkawy A., Samir R., El-Kassas M. (2022). Fibrosis regression following hepatitis C antiviral therapy. World J. Hepatol..

[28] Degos F., Perez P., Roche B., Mahmoudi A., Asselineau J., Voitot H., Bedossa P. (2010). FIBROSTIC study group: Diagnostic accuracy of FibroScan and comparison to liver fibrosis biomarkers in chronic viral hepatitis: A multicenter prospective study (the FIBROSTIC study). J. Hepatol..

[29] Leuștean A., Popescu C., Nichita L., Tilișcan C., Aramă V. (2021). Dynamics of APRI and FIB-4 in HCV cirrhotic patients who achieved SVR after DAA therapy. Exp. Ther. Med..

[30] Leuștean A., Olariu M.C., Mihai N., Tilișcan C., Molagic V., Duport-Dodot I., Stratan L.M., Aramă S.S., Aramă V. (2024). Regression of liver fibrosis in HCV cirrhotic patients treated with interferon-free therapies. Farmacia.

[31] Tawazun Health Interpretation of FibroScan. https://tawazunhealth.com/blog/the-path-to-liver-health-understanding-liver-stiffness/.

[32] Ridgeway G., McCaffrey D., Morral A., Griffin B.A., Burgette L., R Twang (c) Twang: Toolkit for Weighting and Analysis of Nonequivalent Groups (2017). R Package, Version 1.5. https://CRAN.R-project.org/package=twang.

[33] Poynard T., McHutchison J., Manns M., Trepo C., Lindsay K., Goodman Z., Ling M.H., Albrecht J. (2002). Impact of pegylated interferon alfa-2b and ribavirin on liver fibrosis in patients with chronic hepatitis C. Gastroenterology.

[34] George S.L., Bacon B.R., Brunt E.M., Mihindukulasuriya K.L., Hoffmann J., Di Bisceglie A.M. (2009). Clinical, virologic, histologic, and biochemical outcomes after successful HCV therapy: A 5-year follow-up of 150 patients. Hepatology.

[35] Abu-Freha N., Abu-Kosh O., Yardeni D., Ashur Y., Abu-Arar M., Yousef B., Monitin S., Weissmann S., Etzion O. (2023). Liver fibrosis regression and associated factors in HCV patients treated with direct-acting antiviral agents. Life.

[36] Yoo H.W., Park J.Y., Kim S.G., Jung Y.K., Lee S.H., Kim M.Y., Jun D.W., Jang J.Y., Lee J.W., Kwon O.S. (2022). Regression of liver fibrosis and hepatocellular carcinoma development after HCV eradication with oral antiviral agents. Sci. Rep..

[37] Trivedi H.D., Curry M.P., Lai M. (2021). Reply to: The presence of diabetes impacts liver fibrosis and steatosis by transient elastography in a primary care population. Ann. Hepatol..

[38] Sato S., Kawai H., Sato S., Iwasaki H., Omori M., Kita Y., Ikeda Y., Awatsu T., Murata A., Taniguchi G. (2022). Hypertension and diabetes mellitus are associated with high FIB-4 index in a health checkup examination cohort without known liver disease. BMC Gastroenterol..

[39] Ignat M.D., Balta A.A.S., Barbu R.E., Draganescu M.L., Nechita L., Voinescu D.C., Nechita A., Stefanopol I.A., Busila C., Baroiu L. (2024). Antiviral Therapy of Chronic Hepatitis B Virus between Present and Future. J. Clin. Med..

[40] Balta A.A.S., Ignat M.D., Barbu R.E., Baroiu L., Moroianu L.A., Lutenco V., Bulza V., Patriciu M., Dumitru C., Debita M. (2025). HBV, HCV, and HDV Triple-Infection—A Therapeutic Challenge. Diseases.

[41] Jeong D., Wong S., Karim M.E., Manges A.R., Makuza J.D., Bartlett S.R., Velásquez García H.A., Luster D., Adu P.A., Binka M. (2023). Treatment of HCV with direct-acting antivirals on reducing mortality related to extrahepatic manifestations: A large population-based study in British Columbia, Canada. Lancet Reg. Health–Am..

[42] Moon A.M., Green P.K., Rockey D.C., Berry K., Ioannou G.N. (2020). Hepatitis C eradication with direct-acting anti-virals reduces the risk of variceal bleeding. Aliment. Pharmacol. Ther..

[43] El-Serag H.B., Duong H., Luster M., Kanwal F., Hill D.D., Burroughs M., Hernandez C., Haber B.A., Larsen L.M., Marcinak J.F. (2025). Risk of Hepatocellular Cancer in U.S. Patients with Compensated Cirrhosis Treated with Direct-Acting Antivirals Versus Interferon. Aliment. Pharmacol. Ther..

